# Identification of Self-Incompatibility Related Genes in Sweet Cherry Based on Transcriptomic Analysis

**DOI:** 10.3390/biology14091125

**Published:** 2025-08-25

**Authors:** Chen Feng, Chuanbao Wu, Jing Wang, Wei Wang, Guohua Yan, Yu Zhou, Kaichun Zhang, Xiaoming Zhang, Xuwei Duan

**Affiliations:** 1 Institute of Forestry and Pomology, Beijing Academy of Agriculture and Forestry Sciences, Beijing 100093, China; fengclgs@163.com (C.F.); cb19902010@163.com (C.W.); sduwj@126.com (J.W.); wangw@baafs.net.cn (W.W.); bigjohn6524@hotmail.com (G.Y.); zhouyu05332@126.com (Y.Z.); kaichunzhang@126.com (K.Z.); 2 Key Laboratory of Biology and Genetic Improvement of Horticultural Crops (North China), Ministry of Agriculture and Rural Affairs, Beijing 100093, China; 3 Beijing Engineering Research Center for Deciduous Fruit Trees, Beijing 100093, China

**Keywords:** ‘Tieton’, transcription factor, self-incompatible mechanism, gene expression pattern, Co-expression analysis

## Abstract

Most sweet cherry varieties exhibit typical gametophytic self-incompatibility (GSI) characteristics, necessitating careful configuration of pollination trees to ensure adequate yields. This requirement increases the costs associated with orchard labor, management, and other related expenses. Consequently, cultivating and developing sweet cherry cultivars with self-compatibility can effectively address these challenges. Research into the molecular mechanisms underlying GSI formation can provide vital theoretical support and genetic resources for breeding self-compatible sweet cherries. In this study, we assessed the fruit set rates of ‘Tieton’ following both self- and cross-pollination. Additionally, we conducted a transcriptome analysis of the ‘Tieton’ style (which includes the stigma) at 0, 12, 24, and 48 h post-pollination to identify key genes involved in the self-incompatibility process of sweet cherries. The results indicated that the self-fruiting rate of ‘Tieton’ was significantly lower than that of cross-pollination. We identified a total of 8148 differentially expressed genes (DEGs) through transcriptome analysis, with KEGG pathway analysis revealing that the plant-pathogen interaction, plant hormone signal transduction, and plant MAPK signaling pathways were primarily involved in sweet cherry GSI. Furthermore, we identified 13 core transcription factors (TFs) based on their differential expression patterns, including three ERFs, three NACs, three WRKYs, two HD-ZIPs, one RAV, and one MYB. Co-expression analysis identified 132 core DEGs significantly associated with these TFs. Ultimately, this study provides initial insights into the key genes within the sweet cherry GSI network, laying a theoretical foundation and offering genetic resources for the future molecular design breeding of new self-compatible varieties.

## Simple Summary

Plant self-incompatibility involves the recognition of ‘self’ and ‘non-self’ pollen by plants, primarily governed by genes located at the S-locus. However, further research is necessary to explore whether additional non-S-locus genes play a role in regulating self-incompatibility. Currently, there is limited research specifically focusing on self-incompatibility in sweet cherry. In the present study, we conducted a transcriptomic analysis of the style of the ‘Tieton’ variety following both self- and cross-pollination. Through this analysis, we identified several non-S-locus genes that may be involved in the regulation of self-incompatibility in sweet cherries. These findings are anticipated to enhance our understanding of the self-incompatibility mechanism in sweet cherries and provide a foundation for breeding self-compatible sweet-cherry varieties.

## 1. Introduction

Self-incompatibility is a reproductive isolation mechanism that flowering plants have evolved to adapt to varying external conditions and preserve the genetic diversity of their species. This mechanism allows plants to reject their own pollen while accepting pollen from different individuals, facilitating double fertilization. Self-incompatibility can be categorized into sporophytic self-incompatibility (SSI) and gametophytic self-incompatibility (GSI) [1,2,3]. Most fruit trees in the Rosaceae family exhibit S-RNase-mediated GSI, with their self-incompatibility phenotype predominantly regulated by the S-locus [4,5,6,7].

Sweet cherry (*Prunus avium* L.) is among the earliest-maturing deciduous fruit trees to flourish in northern China, and consumers highly appreciate its fruits for their vibrant colors, sweet flavor, and rich nutritional value. Current statistics indicate that the area dedicated to sweet cherry cultivation in China has reached 256,400 ha [8]. Most sweet cherry varieties display typical GSI characteristics, necessitating the presence of other varieties as pollination partners to ensure optimal yields [9]. Research into the mechanism of GSI in sweet cherry started later than in apples or pears, revealing that the GSI phenotype is governed by the S-locus located on chromosome 6 [4,5,7,9]. Combinations of two S-locus alleles form S-genotypes of different varieties; for example, ‘Tieton’ has the genotype *S_3_S_9_*, while ‘Rainier’ has *S_1_S_4_*. Each variety can produce two different S-haplotype pollens; for instance, ‘Tieton’ can produce either *S_3_* or *S_9_* pollen [10]. During pollination, pollen genotypes are considered unaffiliated when they match either genotype of the style, while they are deemed affiliated otherwise. Multiple *F-box* genes known as *S-locus F-box brothers* (*SFBB*) are present on the S-locus of apples and other members of the Rosaceae family; however, only a single *S-haplotype-specific F-box* gene has been identified on the sweet cherry S-locus [11,12,13,14]. This unique characteristic suggests that the molecular mechanism underlying GSI in cherries differs considerably from that in apples, pears, and other fruit trees (Rosaceae).

Three types of self-compatibility (SC) mutations have been identified in sweet cherry: base deletion of the *SFB4* gene, which results in premature translation termination; complete deletion of the *SFB3* gene from the genome; and a 1000 bp sequence insertion within the coding region of the *SFB5* gene [12,15,16,17,18]. The mutation of *the SFB5 gene in the* SC variety ‘Kronio’ occurred naturally [16,19]. Additionally, mutant variants of the *SFB3* and *SFB4* genes were developed through X-ray mutation during SC breeding of sweet cherries. From these cultivars, several SC varieties, including ‘Lapins (*S_1_S_4_′*)’, ‘Yanyang (*S_3_S_4_′*)’, and ‘Sweetheart (*S_3_S_4_′*)’, were produced through cross-breeding [20].

Further studies have indicated that sweet cherry SFB proteins play a crucial role in recognizing and protecting the self S-RNase from degradation by the general inhibitor (GI), a class of F-box proteins that can eliminate self S-RNase via the ubiquitination pathway mediated by the 26S proteasome. When SFB protein functions are lost, the GI can degrade its own S-RNase, leading to self-compatibility in sweet cherries [21,22,23,24,25]. The interaction between stigma S-RNase proteins at the S-locus and different F-box proteins in pollen is a key research area for understanding the molecular mechanisms of sweet cherry GSI. However, studies on the types and functions of non-S-locus genes are relatively scarce. Some research has indicated [26,27] that SC in ‘Cristobalina’ is associated with a non-S-locus gene. Specifically, a transposon-like sequence in the promoter region of the putative *M locus-encoded GST* (*MGST*) downregulated *MGST* expression levels, potentially contributing to the SC of this cultivar. However, the exact molecular mechanism remains unclear. Therefore, there is a pressing need to explore non-S-locus genes further and analyze the specific molecular mechanisms involved in sweet cherry GSI.

The early flowering period of sweet cherries often exposes them to extreme weather conditions, such as late frosts, spring cold spells, high winds, and sandstorms, which can impede insect pollination [9,28]. As a result, artificial pollination is frequently required to ensure a satisfactory fruiting rate, consequently increasing the management costs associated with orchards. Cultivating SC varieties is an effective approach to simplify orchard management and reduce labor requirements. However, current SC resources for sweet cherries are limited, and the selection and breeding of SC varieties through traditional cross-breeding methods can be challenging. This study aims to explore key genes associated with GSI in sweet cherries using transcriptome sequencing and other technologies. By utilizing self-pollinated and cross-pollinated ‘Tieton’ style (containing stigma) as test materials, we seek to clarify the molecular mechanisms involved in the formation of GSI. Ultimately, this research aims to provide valuable genetic resources and theoretical guidance for developing SC varieties.

## 2. Results

### 2.1. Pollen Morphology and Activity Analysis of ‘Tieton’ and ‘Rainier’

**Figure 1:**
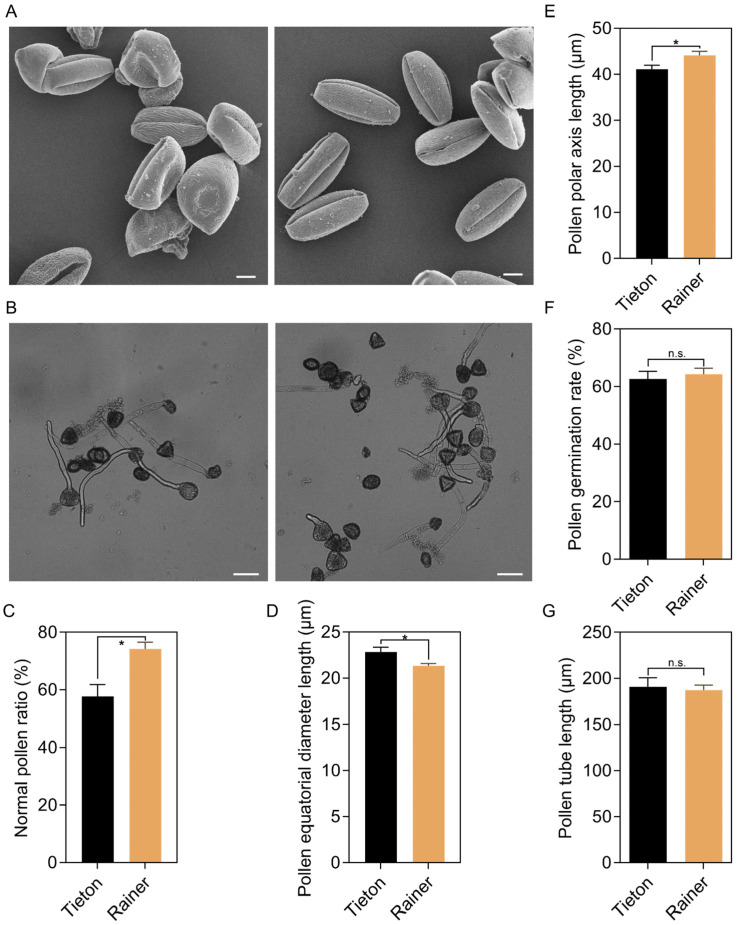
Morphology and activity analysis of ‘Tieton’ and ‘Rainier’ pollens. (**A**) Observation of pollen morphology, Bar = 100 μm; (**B**) Observation of pollen germination, Bar = 50 μm; (**C**) Normal pollen ratio, data was obtained from three different visual fields; (**D**) Pollen equatorial diameter length, μm, *n* = 20; (**E**) Pollen polar axis length, μm, *n* = 20; (**F**) Pollen germination rate, data was obtained from three different visual fields; (**G**) Pollen tube elongation after 2 h germination, μm, data was obtained from three different visual fields. All quantitative data are presented as means ± SDs. Bars indicate SD. Asterisks indicate significant differences (* *p* < 0.05, n.s., no significance, Student’s *t*-test).

Before conducting self- and cross-pollination of the ‘Tieton’ sweet cherry, we examined the pollen morphology of both ‘Tieton’ and ‘Rainier’ using scanning electron microscopy (Figure 1A). Our findings indicated that the normal pollen ratio was 57.70% for ‘Tieton’ and 74.26% for ‘Rainier’ (Figure 1C). Additionally, we measured the average polar axis length of the pollen grains to be 41.09 ± 4.00 μm for ‘Tieton’ and 44.11 ± 3.88 μm for ‘Rainier’, while the average equatorial diameter length was recorded as 22.83 ± 2.26 μm for ‘Tieton’ and 21.35 ± 1.11 μm for ‘Rainier’ (Figure 1D,E).

Following this, the pollen germination rate was assessed. The results (Figure 1B,F) demonstrated that the pollen germination rates for both ‘Tieton’ and ‘Rainier’ exceeded 60%, with an average pollen tube elongation of approximately 190 μm (Figure 1G). This indicates that pollen vigor was high, making it suitable for subsequent pollination experiments.

The self-incompatibility (SI) analysis of the ‘Tieton’ variety was conducted using ‘Tieton’ as the female parent and both ‘Tieton’ and ‘Rainier’ as the male parents for self- and cross-pollination, respectively. One and a half months after pollination, we analyzed the fruit set rates and discovered that the self-fruiting set rate for ‘Tieton’ was a mere 2%, whereas the rate for cross-pollination reached 56% (Table 1). These findings confirm that ‘Tieton’ sweet cherry exhibits SI characteristics.

### 2.2. Transcriptome Analysis

**Figure 2:**
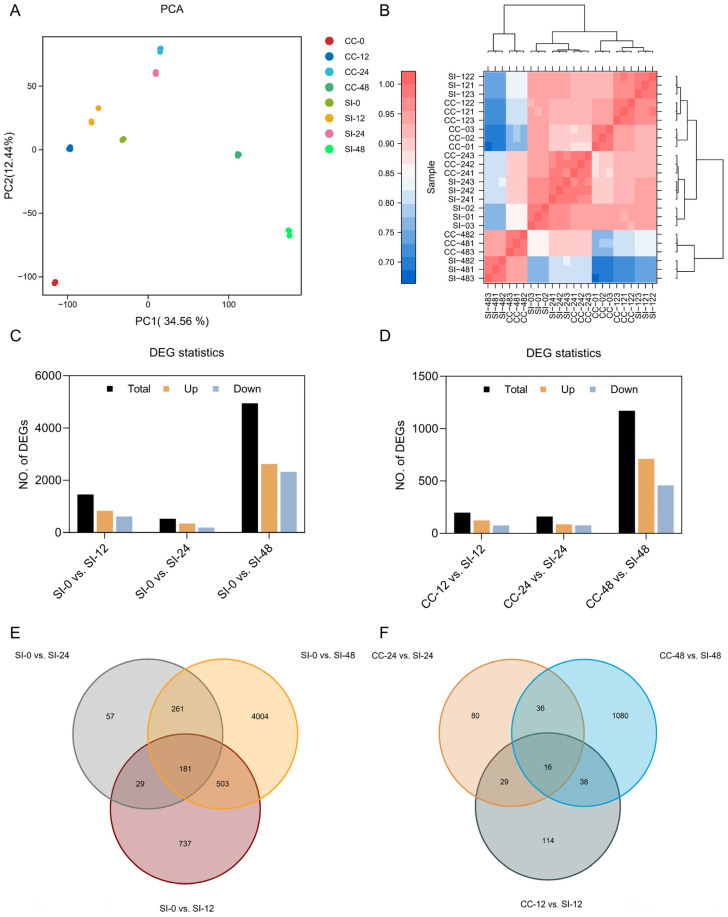
Transcriptome analysis of style samples at intervals of 0 h, 12 h, 24 h, and 48 h following both self- and cross-pollination. (**A**) PCA of the transcriptomes; (**B**) The correlation among samples was evaluated using Pearson’s correlation coefficients, with values representing the strength of the relationships between samples; (**C**) Statistics of DEGs among SI-0 vs. SI-12, SI-0 vs. SI-24, and SI-0 vs. SI-48 comparison groups; (**D**) Statistics of DEGs among CC-12 vs. SI-12, CC-24 vs. SI-24, and CC-48 vs. SI-48 comparison groups; (**E**) Venn diagrams of DEGs among SI-0 vs. SI-12, SI-0 vs. SI-24, and SI-0 vs. SI-48 comparison groups; (**F**) Venn diagrams of DEGs among CC-12 vs. SI-12, CC-24 vs. SI-24, and CC-48 vs. SI-48 comparison groups.

To elucidate the molecular mechanism of SI in ‘Tieton’, we conducted a transcriptomic analysis of style samples at intervals of 0, 12, 24, and 48 h following both self- and cross-pollination. We obtained a total of 152.66 GB of clean data (Supplementary Table S1), and the gene Fragments Per Kilobase of transcript per million mapped reads (FPKM) values ranged from 10^−2^ to 10^4^ (Supplementary Figure S1). Principal component analysis (PCA) and correlation analysis of the samples confirmed their reproducibility and allowed for clear differentiation among the various groups, thereby ensuring the reliability and precision of our findings (Figure 2A,B).

Statistical analysis of differentially expressed genes (DEGs) revealed that (Figure 2C), compared to self-pollination at 0 h (SI-0), there were 1450 DEGs in SI-12, comprising 831 upregulated and 619 downregulated genes. In SI-24, 528 DEGs were identified, of which 340 genes were upregulated and 188 genes were downregulated. Notably, SI-48 exhibited a significant increase, totaling 4949 DEGs, which included 2625 upregulated and 2324 downregulated genes. We also assessed the DEGs resulting from the various pollination methods (Figure 2D). When comparing cross-pollination at 12 h (CC-12) with SI-12, we identified 197 DEGs, including 123 that were upregulated and 74 that were downregulated. For the comparison of CC-24 vs. SI-24, 161 DEGs were detected, of which 85 were upregulated and 76 downregulated. Finally, in comparison with CC-48, SI-48 displayed 712 upregulated and 458 downregulated DEGs. These DEGs may play distinct roles in the SI mechanism of sweet cherries (Supplementary Table S2).

Venn analysis of the differential comparison groups (Figure 2E) indicated that the SI-0 vs. SI-48 comparison yielded the highest number of specific DEGs, totaling 4004 genes. Additionally, we identified 181 core DEGs common to the comparisons of SI-0 vs. SI-12, SI-0 vs. SI-24, and SI-0 vs. SI-48, suggesting that these genes may be significantly associated with sweet cherry SI. Furthermore, Venn analysis of various pollination methods (Figure 2F) revealed that the CC-48 vs. SI-48 comparison group contained the largest number of specific DEGs, amounting to 1080 genes. In this context, 16 core DEGs were found across the CC-12 vs. SI-12, CC-24 vs. SI-24, and CC-48 vs. SI-48 comparison groups, indicating their likely involvement in sweet cherry SI.

### 2.3. GO Term and KEGG Pathway Analysis of DEGs

**Figure 3:**
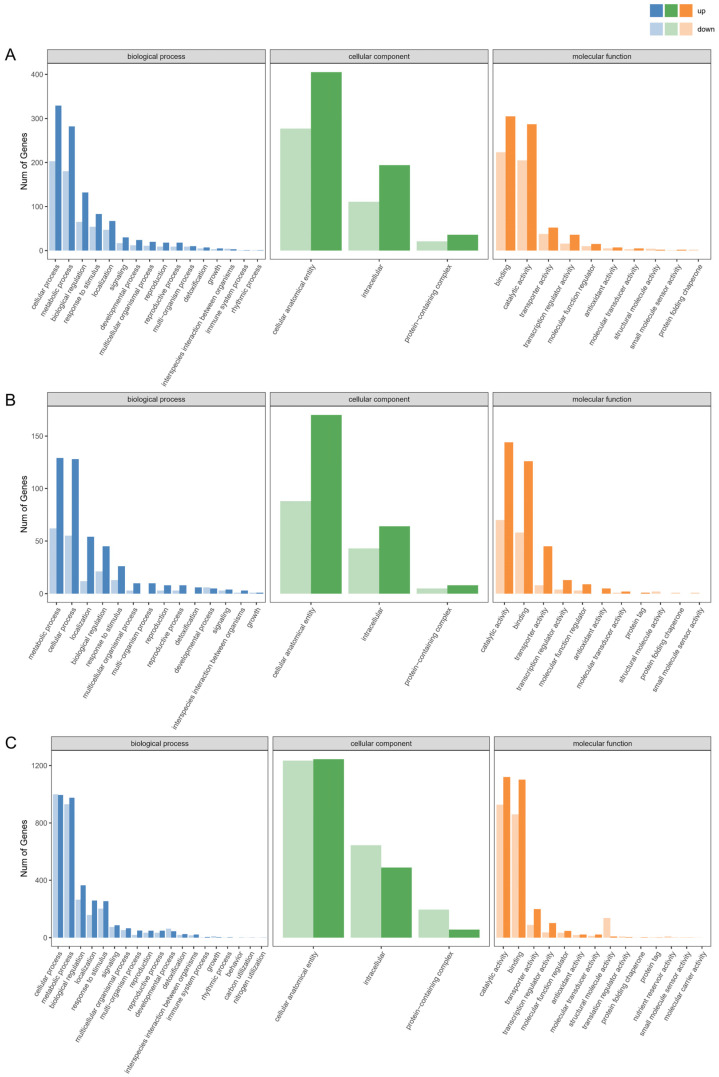
GO enrichment analysis of DEGs across distinct comparison groups. (**A**) SI-0 vs. SI-12; (**B**) SI-0 vs. SI-24; (**C**) SI-0 vs. SI-48.

GO term enrichment analysis effectively identifies DEGs that are significantly associated with specific biological functions. In this study, the DEGs were categorized into three main ontological categories: biological process, cellular component, and molecular function (Figure 3). Within the SI-0 vs. SI-12 comparison group, the biological process category exhibited strong enrichment for cellular and metabolic processes, accounting for 532 and 462 DEGs, respectively. Additionally, significant representations were found in biological regulation, response to stimuli, and localization, encompassing 197, 137, and 114 DEGs, respectively. These findings suggest that these biological processes play crucial roles in SI at 12 h post-pollination. Regarding cellular components, the two most enriched subgroups were cellular anatomical entities and intracellular, with 682 and 305 DEGs identified, respectively. The two subgroups with the highest enrichment of molecular functions were binding and catalytic activity, which had 528 and 492 enriched DEGs, respectively. In the SI-0 vs. SI-24 comparison, the metabolic process subgroup showed the highest number of DEG in the biological process, totaling 191, followed by cellular process, localization, biological regulation, and response to stimulus. Notably, the cellular component subgroup distributions mirrored those of the SI-0 vs. SI-12 comparison, although the number of DEGs varied. In the molecular function category, the subgroup with the highest DEG enrichment was catalytic activity, which comprised 214 genes. The same trend persisted in the SI-0 vs. SI-48 comparison group, where cellular and metabolic processes remained predominant among the biological process subgroups. Furthermore, GO analysis was conducted on the comparison groups involving cross- and self-pollination (Supplementary Figure S2). The top five biological process subgroups were consistent across the CC-12 vs. SI-12, CC-24 vs. SI-24, and CC-48 vs. SI-48 comparisons, albeit with differing DEG counts. These results indicate that SI in the ‘Tieton’ cultivar is primarily linked to cellular and metabolic processes, highlighting that fundamental biological processes do not significantly fluctuate with prolonged pollination duration.

**Figure 4:**
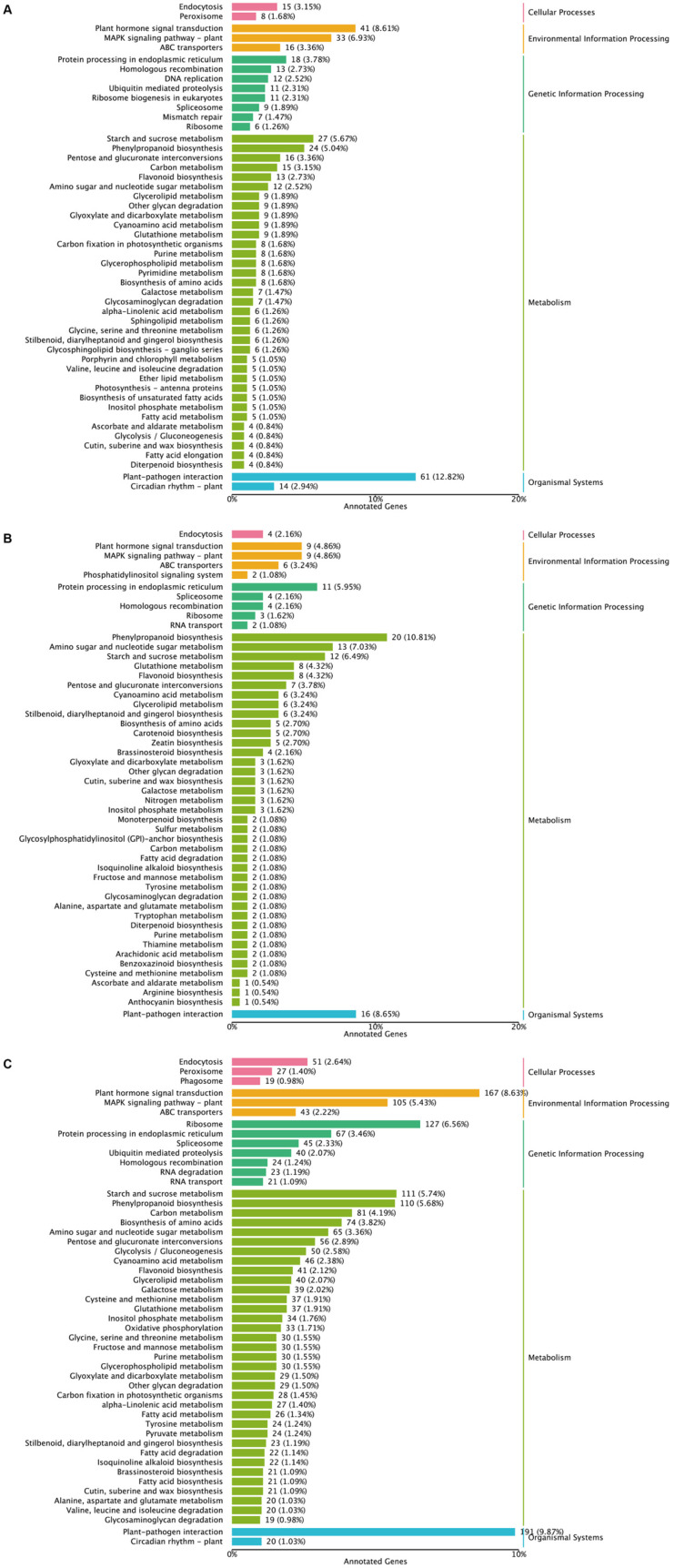
KEGG pathway category analysis of DEGs across distinct comparison groups. (**A**) SI-0 vs. SI-12; (**B**) SI-0 vs. SI-24; (**C**) SI-0 vs. SI-48.

**Figure 5:**
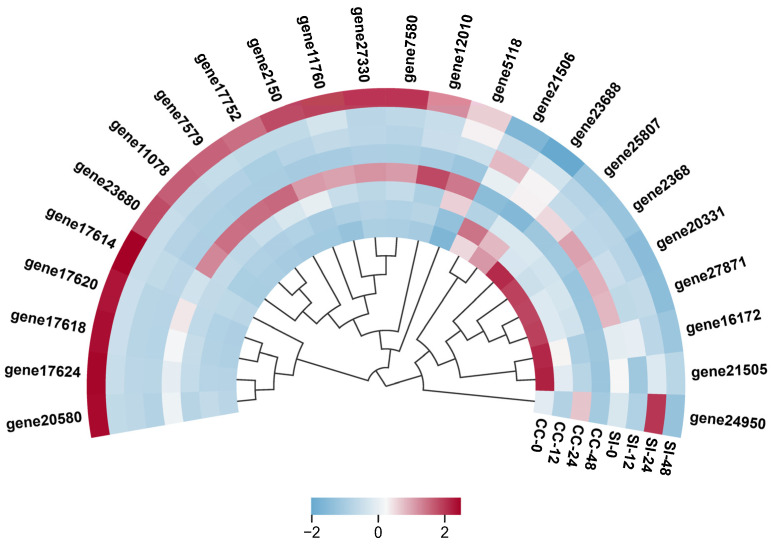
Heatmap of 20 DEGs in the phenylpropanoid biosynthesis pathway. Values are means of three biological replicates.

To gain deeper insight into the role of DEGs in ‘Tieton’ following various pollination methods, we conducted KEGG pathway enrichment analysis of DEGs across distinct comparison groups (Figure 4). Our findings revealed that the metabolic pathway exhibited the highest number of DEGs across all comparison groups. Notably, a comparative analysis indicated that in relation to SI-0, the groups SI-12, SI-24, and SI-48 displayed significant enrichment of DEGs in three key pathways: plant-pathogen interaction, plant hormone signal transduction, and plant MAPK signaling pathway. This suggests that these pathways and associated DEGs play essential roles in the different outcomes of the pollination processes in ‘Tieton’. In the SI-0 vs. SI-12 comparison, we identified 476 DEGs that were enriched in 112 metabolic pathways. Specifically, 61 DEGs were associated with the plant-pathogen interaction pathway, 41 DEGs were linked to the plant hormone signal transduction pathway, and 33 DEGs were identified in the plant MAPK signaling pathway. In the SI-0 vs. SI-24 comparison group, 185 DEGs were enriched in 76 metabolic pathways; of these, 16 DEGs were related to the plant-pathogen interaction pathway, 9 DEGs to the plant hormone signal transduction pathway, and another 9 DEGs to the plant MAPK signaling pathway. Additionally, 20 DEGs were enriched in the phenylpropanoid biosynthesis pathway (Figure 5), indicating the potential involvement of this pathway in SI after 24 h of self-pollination. The SI-0 vs. SI-48 comparison revealed a more extensive response, with 1935 DEGs enriched in 127 metabolic pathways, of which 191 DEGs were associated with the plant-pathogen interaction pathway, 167 DEGs with the plant hormone signal transduction pathway, and 105 DEGs with the plant MAPK signaling pathway. Furthermore, we observed that 127 DEGs were enriched in the ribosome pathway, suggesting their potential role in sweet cherry SI. Notably, the plant-pathogen interaction pathway was also enriched in the CC-12 vs. SI-12, CC-24 vs. SI-24, and CC-48 vs. SI-48 comparison groups, highlighting the consistent involvement of this pathway across various treatment conditions (Supplementary Figure S3).

### 2.4. Screening of Candidate Genes Related to SI in ‘Tieton’

The findings from the KEGG enrichment analysis indicate that most comparison groups exhibited significant enrichment of DEGs within the plant-pathogen interaction, plant hormone signal transduction, and MAPK signaling pathways related to plants. Notably, the plant-pathogen interaction pathway demonstrated the highest incidence of DEGs, totaling 231, which predominantly consisted of receptor-like proteins, receptor-like protein kinases, serine/threonine-protein kinases, calcium/calmodulin-regulated receptor-like kinases, calcium-binding proteins, pathogenesis-related proteins, and WRKY transcription factors (Supplementary Table S3). Among the DEGs identified, three genes, *RADIALIS-like 5* (*gene2270*), *WRKY33* (*gene19686*), and *WRKY40* (*gene16143*), were significantly upregulated at 12, 24, and 48 h after self-pollination, with peak upregulation observed at 48 h in SI-48. Specifically, the expression levels of these genes were 4.97-, 5.91-, and 7.35-fold higher than those in the control group (SI-0). Additionally, *RADIALIS-like 5* showed substantial upregulation in SI-12 compared to CC-12, with a fold change of 1.67. Meanwhile, *WRKY33* and *WRKY40* were also elevated in SI-48 relative to CC-48, with fold changes of 1.07 and 1.33, respectively. These findings suggest that *RADIALIS-like 5*, *WRKY33*, and *WRKY40* are pivotal genes influencing pollen recognition mechanisms in sweet cherry.

Furthermore, 10 additional genes were identified as significantly upregulated at 24 and 48 h following self-pollination. Among these, *tyrosine-sulfated glycopeptide receptor 1* (*gene10914*) emerged as the most highly upregulated, exhibiting fold changes of 2.77 and 5.86 in SI-24 and SI-48, respectively, compared to SI-0. *Receptor-like protein 21* (*gene23680*) was also notably up-regulated in SI-48, exhibiting a 3.82-fold increase compared to SI-0. *WRKY22* (*gene5772*) had fold changes of 1.06 and 2.51 in SI-24 and SI-48, respectively, compared to SI-0. Notably, the *serine/threonine-protein kinase-like protein CCR4* (*gene19447*) was upregulated by 1.72-fold in SI-24, placing it second only to *gene10914*. Additionally, a new gene, *Prunus_avium_newGene_83*, was identified within this metabolic pathway as potentially involved in the pollen recognition process of sweet cherry, with fold increases of 1.11 and 2.21 in SI-24 and SI-48, respectively. Collectively, these results further support the notion that self-pollination recognition mechanisms in sweet cherry parallel the plant’s response to external pathogen challenges.

**Figure 6:**
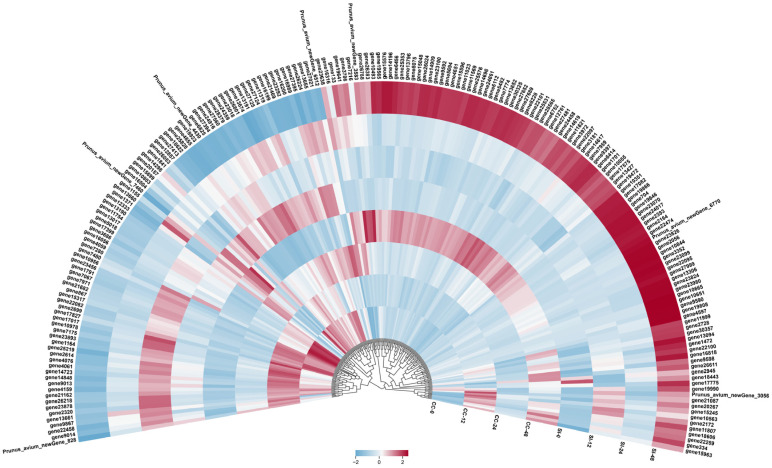
Heatmap of 194 DEGs in the plant hormone signal transduction pathway. Values are means of three biological replicates.

A total of 194 DEGs were identified in the plant hormone signal transduction pathway, primarily involving response factors, functional genes, and transcription factors (TFs) associated with the auxin pathway (Figure 6; Supplementary Table S4). Additionally, several receptor-like proteins and serine/threonine-protein kinases were detected, suggesting that auxin is the primary plant hormone involved in SI in sweet cherries. Significant expression changes were observed in 10 genes at 24 and 48 h post-self-pollination compared to the control group (SI-0). Among these, *GH3.6* (*gene6112*) exhibited the highest upregulation, with expression levels 2.20 and 7.11 times greater than that of SI-0 at SI-24 and SI-48, respectively. *GH3.6* was also upregulated 1.37-fold in SI-24 relative to CC-24. In contrast, *AUX22* (*gene13190*) was significantly downregulated in both SI-24 and SI-48, with expression levels decreasing by −1.39 and −5.60 times compared to SI-0, respectively, and a 2.06-fold decrease in SI-48 compared to CC-48. Furthermore, *histone deacetylase* (*gene7280*) also showed significant decreases in both SI-24 and SI-48, with reductions of −1.02- and −1.20-fold of SI-0, respectively, suggesting that histone deacetylation may affect pollination affinity in sweet cherry. Additionally, a putative *MYB* gene (*gene19641*) was found to be upregulated by 1.14- and 1.46-fold in SI-24 and SI-48, respectively. A total of 50 DEGs were identified in SI-48 compared to CC-48 in the plant hormone signal transduction pathway. Beyond auxin-related genes, *TIFY* (*gene13306* and *gene21087*), *SPL* (*gene2164*), and *F-box* (*gene13094* and *gene4076*) genes were identified, all of which were upregulated, except for *gene4076*, which was significantly downregulated. These findings further suggest that, in addition to auxin, several genes related to flower development may play essential roles in SI in sweet cherries.

A total of 126 DEGs were identified in the plant MAPK signaling pathway, primarily comprising calcium-binding proteins and serine/threonine-protein kinases (Supplementary Table S5). This pathway also included *WRKYs*, specifically *WRKY33* and *WRKY22*, suggesting their involvement in the pollination process of sweet cherry through various regulatory pathways. The *phytosulfokine receptor 1* (*gene8229*) was upregulated in SI-12, SI-24, and SI-48, showing increases of 1.27-, 1.25-, and 3.29-fold, respectively, compared to SI-0. *Endochitinase 1* (*gene5625*) exhibited a significant increase 24 h after self-pollination, with expression increasing 5.09-fold in SI-48. Furthermore, 25 DEGs were identified when comparing SI-48 cells to CC-48 cells. Among these, *WRKY72A* (*gene5959*) and *WRKY75* (*gene2160*) were notably enriched in this pathway, with expression levels increasing by 5.54- and 1.83-fold, respectively. This suggests that these two *WRKYs* may play a role in sweet cherry SI by regulating MAPK signaling. Additionally, several pathogenesis-related proteins and protein phosphatase 2Cs, including *gene10651*, *gene10665*, and *gene3352*, were enriched in this pathway, further indicating that sweet cherry SI is intricate and involves multiple pathways.

### 2.5. Transcription Factor Analysis

**Figure 7:**
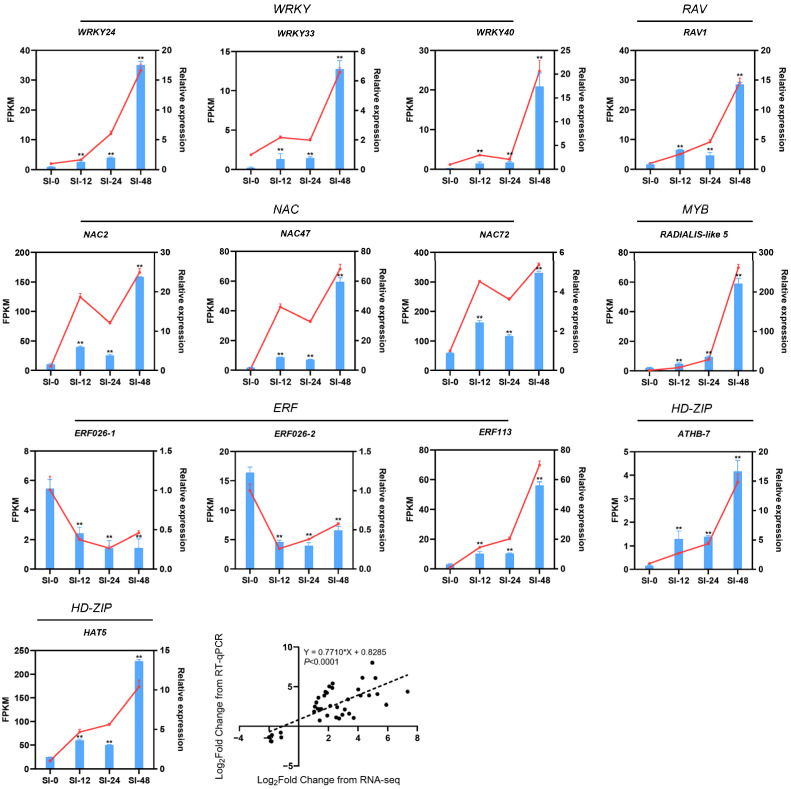
Expression pattern of 13 TFs. Data are means ± SDs of three replicates. Bars indicate SD. Asterisks indicate significant differences compared to SI-0 (** *p* < 0.01, Student’s *t*-test). The blue bars represent RNA-seq data, while the red line indicates RT-qPCR data.

Transcription factor prediction analysis based on 181 core DEGs identified a total of 13 TF genes, which included three *ERFs*, three *NACs*, three *WRKYs*, two *HD-ZIPs*, one *RAV*, and one *MYB*. Except for the two *ERF* genes (*ERF026-1* and *ERF026-2*), the expression levels of the remaining 11 TFs progressively increased during self-pollination, peaking at 48 h (Figure 7 and Supplementary Table S2). Among these, *WRKY40* exhibited the highest fold-change in expression. *WRKY33* and *WRKY24* were the next most highly expressed TFs, with the expression of *WRKY24* at SI-48 being 5.31-fold greater than that at SI-0. *NAC47* also demonstrated an upward trend in expression throughout the SI, peaking at 48 h with a 5.18-fold increase compared to that at 0 h. *NAC2* and *NAC72* displayed similar expression patterns to *NAC47*, with both reaching their highest levels at SI-48, which were 4.02-fold and 2.60-fold increases over SI-0, respectively. The *HD-ZIP gene ATHB-7* showed a significant increase at SI-12, reaching 2.95-fold of SI-0, and continued to rise to 3.11-fold and 4.75-fold at SI-24 and SI-48, respectively. Conversely, the other *HD-ZIP* gene, *HAT5*, was upregulated to 3.33-fold of SI-0 only at SI-48. Interestingly, the expression of two *ERFs*, *ERF026-1* and *ERF026-2*, gradually decreased with an increase in self-crossing time. Notably, the decline in expression of *ERF026-2* was more pronounced than that of *ERF026-1*, with a decrease of −1.84 and −2.00 times at SI-12 and SI-24 relative to 0 h, respectively.

To confirm the accuracy of the gene expression results obtained from RNA-seq, we selected the aforementioned 13 TF genes for RT-qPCR analysis. The results (Figure 7) demonstrated a strong correlation between the expression patterns observed through RT-qPCR and those identified via RNA-seq (*p* < 0.0001), thereby confirming the reliability of our RNA-seq data.

### 2.6. Co-Expression Network Analysis

**Figure 8:**
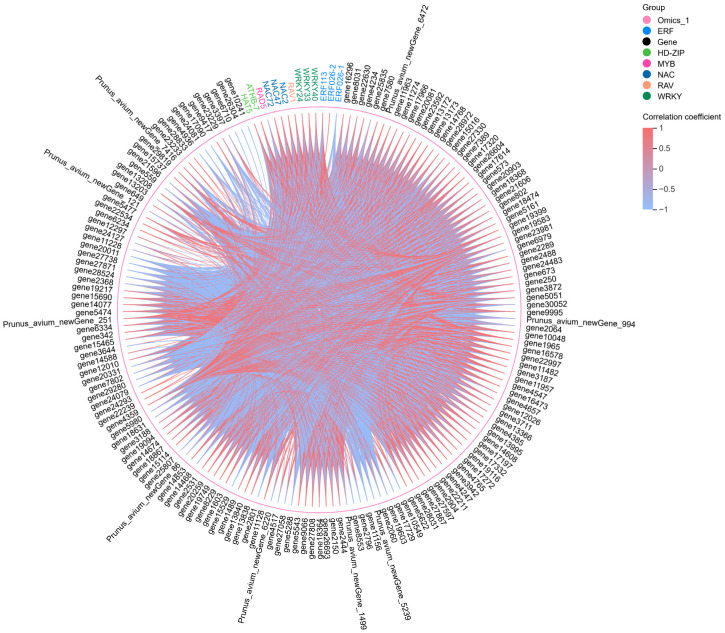
The chord diagram of 13 TFs and core DEGs. 13 TF genes were marked with different colors, including 3 *NACs* (*NAC2*, *NAC47*, and *NAC72*), 3 *WRKYs* (*WRKY24*, *WRKY33*, and *WRKY40*), 3 *ERFs* (*ERF026-1*, *ERF026-2*, and *ERF113*), 2 *HD-ZIPs* (*ATHB-7* and *HAT5*), 1 *RAV* (*RAV1*), and 1 *MYB* (*RAD5*). Core DEGs were colored black. Red lines between different genes mean positive correlation, while blue lines indicate negative correlation.

To further elucidate the molecular mechanisms underlying SI in sweet cherry, a chord diagram was generated using the FPKM values of the core DEGs and 13 TF genes (Figure 8). A comprehensive analysis was conducted to evaluate the correlations between these 13 TF genes and the core DEGs, focusing on retaining data exhibiting correlation coefficients exceeding 0.8 and *p*-values below 0.05 (Supplementary Table S6). Notably, *WRKY33* was enriched in the highest number of core DEGs, indicating 104 positive and 22 negative correlations. Among these, the *proline-rich cell wall protein 1-like* (*gene4247*) exhibited the strongest correlation coefficient of 0.98 with *WRKY33*, with its expression levels progressively increasing; in particular, expression in SI-48 was recorded as 5.21 times that of SI-0. In contrast, *vinorine synthase-like* (*gene25807*) displayed a negative correlation with *WRKY33*, with its expression levels decreasing progressively, measured at −1.23, −1.59, and −3.91 times that of SI-0 in SI-12, SI-24, and SI-48, respectively. Furthermore, 101 genes demonstrated significant positive correlations with *WRKY40*, while 22 showed negative correlations. Among these, *SWEET1-like* (*gene26693*) presented the highest positive correlation coefficient of 0.97 with *WRKY40*, with expression levels 3.65-fold higher in SI-48 than in SI-0.

Additionally, a novel gene, *Prunus_avium_newGene_86*, was identified as being significantly negatively correlated with *WRKY40*, revealing expression levels that were −5.20-fold lower than those in SI-0 in SI-48. This suggests a potentially critical role for *Prunus_avium_newGene_86* in sweet cherry SI, with gene ontology (GO) annotation indicating its involvement in the biological process of response to wounding (GO:0009611). A total of 124 DEGs were significantly associated with *ATHB-7*, with 103 exhibiting positive correlations and 21 showing negative correlations. The *glucan endo-1,3-beta-glucosidase* (*gene1489*) attained the highest positive correlation coefficient of 0.96 with *ATHB-7*, peaking at 48 h post self-pollination at an expression level 4.84 times that of SI-0. Furthermore, 23 genes were associated with *ERF026-1*, of which three were positively correlated and 20 were negatively correlated. Among these, *CYP749A22-like* (*gene18631*) exhibited the highest positive correlation coefficient of 0.82, while the *pathogenesis-related protein PR-4-like* (*gene8031*) demonstrated a contrasting correlation coefficient of −0.88, marked by a significant upregulation in SI-48 at a level 7.34 times that of SI-0. These findings suggest that the regulatory network constituted by these core TFs and differentially functional genes is likely to play a pivotal role in sweet cherry SI.

## 3. Discussion

Most fruit trees, including apple, pear, peach, apricot, and cherry, are known to experience S-RNase-based self-pollination disaffinity [29,30,31,32,33]. In this study, our observations on pollen viability demonstrate that pollen is suitable for pollination experiments (Figure 1). We examined the fruit set rate of ‘Tieton’ through self- and cross-pollination, observing a mere 2% success rate for self-pollination compared to 56% for cross-pollination. This confirms that ‘Tieton’ is indeed a self-incompatible sweet cherry variety. Our previous research established that in the self-compatible sweet cherry ‘Lapins’, a mutation in SBF4 prevents the recognition of self S-RNase, leading to the degradation of S-RNase and alleviating its cytotoxic effects, thus allowing pollen tube to continue their growth [25]. However, it remains to be determined whether a similar mechanism occurs in self-incompatible sweet cherry varieties. While existing studies on SI have predominantly focused on S-RNase and SBF [24,25,33], the potential involvement of other non-S factors in regulating SI in sweet cherries has not yet been thoroughly explored. Through transcriptome sequencing in this study, we discovered that DEGs were primarily associated with the plant-pathogen interaction, plant hormone signal transduction, and MAPK signaling pathways in plants. This suggests that these three pathways and their associated DEGs play vital roles in sweet cherry SI.

The process of pollen recognition by plant stigma, known as pollen-stigma interactions, mirrors the mechanisms involved in plant-pathogen interactions [34]. In this study, several receptor-like proteins (RLPs) and receptor-like protein kinases (RLKs) associated with immune signaling recognition within the plant-pathogen interaction pathway were identified using GO and KEGG enrichment analyses. RLPs and RLKs are crucial components of a plant’s capacity to sense external environmental cues and cellular stimuli, and play significant roles in various life processes, including growth and development, reproduction, and immune responses [35,36,37]. Hou et al. [38] revealed a substantial number of upregulated *RLK* genes when comparing self-pollinated to cross-pollinated groups using *Corylus heterophylla* as the model organism. This finding suggests that *RLKs* play a vital role in pollen recognition and rejection processes. Additionally, transcriptome analysis of *Leymus chinensis* stigmas conducted by Zhou et al. [39] identified many differentially expressed *RLK* genes. In our study, we identified a total of 19 differentially expressed *RLKs*, with *proline-rich receptor-like protein kinase PERK8* (*gene4248*) being the most highly upregulated in SI-48, at 5.10-fold higher than that in SI-0. These results further affirm the vital role of RLKs in sweet-cherry SI. Calcium signaling plays a critical role in plant SI. It has been demonstrated that calcium is essential for the normal growth of pollen tubes, and that Ca^2+^ ions also serve as vital messengers mediating the style response to pollen [38,40,41]. In this study, we identified *calcium/calmodulin-regulated receptor-like kinase 1* (*gene20982*) as being differentially expressed during self-pollination in sweet cherries, suggesting that this gene may influence sweet cherry SI.

Plant hormones play a crucial role in the pollination process of flowering plants [42,43,44]. Previous research has demonstrated that auxin is integral in regulating this process. For instance, Chen and Zhao [45] confirmed that free indole-3-acetic acid (IAA) promotes the germination and growth of pollen tubes in tobacco, although the specific mechanism is unclear. A study involving *Torenia fournieri* yielded results consistent with those observed in tobacco, showing that free IAA influences pollen tube growth primarily by affecting pectin and cellulose microfibrils within the tube wall [46]. In this study, DEGs associated with the auxin pathway were identified, including *GH3.6* and *AUX22*. These genes are primarily linked to the regulation of auxin homeostasis and root development. Staswick found that *GH3.6* is involved in the conjugation of free IAA with Asp, which subsequently regulates auxin homeostasis in plants [47]. Additionally, An et al. [48] associated *AUX22* with the development of adventitious roots during the rooting of blueberry cuttings. However, the roles of these two auxin-related genes in the SI mechanisms of sweet cherries warrant further investigation.

MAPK signaling represents a category of signal transduction pathways prevalent in plants and plays a crucial role in defence mechanisms and stress responses. Inhibition of pollen tube growth in SI can be classified as a stress response. Research has shown that SI triggers the expression of the pollen-specific *MAPK* gene *Pr-MPK9-1* in *Papaver rhoeas*, which is implicated in the growth inhibition associated with SI and the initiation of programmed cell death [49]. Studies conducted on *Camellia oleifera* [50] and *Plumbago auriculata* [51] have demonstrated that self-pollination leads to the differential expression of genes related to the MAPK signaling pathway, which is consistent with our findings. Notably, *MAPKKK20* (*gene4131*) was significantly upregulated in SI-48, showing an 8.41-fold increase compared to CC-48 (Supplementary Table S5), suggesting that this gene plays a vital role in sweet cherry SI.

TFs are crucial for regulating many life processes, including plant growth and development. Numerous studies have shown that TFs influence pollen-stigma interactions in plants. In *Arabidopsis thaliana*, WRKY34 and WRKY2 are essential for pollen germination and growth of pollen tubes; their absence results in reduced pollen viability, which negatively impacts pollen tube development [52]. Su et al. [53] demonstrated that ethylene is involved in the SI of *Brassica rapa* by regulating programmed cell death. When the expression of *BrERF012.1* was inhibited using antisense oligonucleotides, the ethylene signaling pathway was disrupted, eliminating the programmed cell death that occurs due to cross-pollination. This finding confirms the involvement of ethylene and ERF in pollen-stigma interactions. Our study identified several differentially expressed WRKY and ERF TFs, including WRKY33, WRKY40, and ERF026, indicating that these TFs may play a role in SI in sweet cherries. Additionally, we identified NAC, HD-ZIP, and MYB TFs, suggesting that a complex network of multiple genes regulates the self-pollination and cross-pollination of sweet cherries. However, the specific mechanisms underlying SI require further investigation.

## 4. Materials and Methods

### 4.1. Plant Materials

Two sweet cherry varieties, ‘Tieton (*S_3_S_9_*)’ (hard flesh and red skin) and ‘Rainier (*S_1_S_4_*)’ (soft flesh and blush skin), were selected as experimental materials. They were cultivated at the Institute of Forestry and Pomology, Beijing Academy of Agriculture and Forestry. Both varieties thrived under the same growth conditions and produced sufficient flowers for further experiments.

### 4.2. Pollen Vitality Identification

Anthers were collected from mature blossom buds of two cultivars, ‘Tieton’ and ‘Rainier’, and kept at room temperature (25 °C) for 24 h. After allowing the pollen to disperse, it was collected for further analysis.

Pollen morphology was examined in both cultivars using a scanning electron microscope (Hitachi S-3400N, Tokyo, Japan). Sample preparation involved carefully collecting dried pollen using a cotton swab, which was then strategically applied to a double-sided conductive adhesive. Following this initial step, the pollen grains were coated with gold sputter coating in an Eiko IB5 Ion Coater to enhance their conductivity and imaging fidelity. Once the gold coating process was completed, the samples were examined. High-resolution images were captured at a magnification of 350× to facilitate a detailed investigation of the morphological characteristics inherent to each pollen.

Pollen vitality was assessed using a reagent kit (BC8430, Beijing Solarbio Science and Technology, Beijing, China) following the manufacturer’s instructions. Briefly, dried pollen was cultured in a liquid pollen medium and incubated at room temperature for 2 h. After incubation, the liquid mixture was carefully transferred onto a glass slide. The pollen germination rate was then assessed using an optical microscope.

### 4.3. Field Pollination Treatments

Two pollination treatments were established: ‘Tieton’ × ‘Tieton’ (self-pollination) and ‘Tieton’ × ‘Rainier’ (cross-pollination). Pollination was conducted on sunny days in early April from 9:00 to 11:00 a.m. To assess fruit set rates, 50 fully developed large flowers were selected for each pollination treatment. Following the pollination process, these flowers were bagged and labeled, and the fruit-set rate was recorded after 45 days. For transcriptome analysis, style samples (which include the stigma) were collected at 0, 12, 24, and 48 h after the different pollination methods. Each pollination treatment included three biological replicates, each comprising 50 styles.

### 4.4. RNA Extraction, Transcriptome Sequencing, and Real-Time Quantitative PCR

Total RNA was extracted from the style samples using the EASYspin Plus Complex Plant RNA Kit (RN5301; Aidlab Biotechnology, Beijing, China). RNA concentration and purity were assessed using a NanoDrop 2000 (Thermo Fisher Scientific, Waltham, MA, USA) and further validated using 1% agarose gel electrophoresis. Sequencing libraries were prepared using the NEBNext UltraTM RNA Library Prep Kit for Illumina (NEB, Ipswich, MA, USA), adhering strictly to the manufacturer’s guidelines, and index codes were incorporated to associate sequences with their respective samples. The comprehensive methodology was documented by Feng et al. [54]. Clean reads were aligned to the reference genome of the sweet cherry cultivar ‘Satonishiki’ (*Prunus avium* Whole Genome Assembly v1.0 & Annotation, https://www.rosaceae.org/species/prunus_avium/genome_v1.0.a1 (accessed on 8 June 2022)) using HISAT. Gene functions were annotated based on multiple databases: NCBI non-redundant protein sequences), Nt (NCBI non-redundant nucleotide sequences), Pfam (Protein family), KOG/COG (Clusters of Orthologous Groups of proteins), Swiss-Prot (a manually curated protein sequence database), KO (KEGG Ortholog database), and GO (Gene Ontology). Gene expression levels were quantified using FPKM (Fragments Per Kilobase of transcript per million mapped reads) [55]. Differentially expressed genes (DEGs) were identified using DESeq2, employing the criteria of |log_2_foldchange| > 1 and *p*-value < 0.05. GO enrichment analysis of the DEGs was conducted using the GOseq R package, while KEGG enrichment analysis was performed using KOBAS 3.0 software.

We selected 13 genes from the differentially expressed genes and verified them using real-time quantitative PCR (RT-qPCR). RT-qPCR was performed in a 20 μL reaction system as described [56,57]. *Actin* was chosen as the internal quantification control for sweet cherry genes. Relative expression levels were measured using the 2^−ΔΔCt^ method [58]. Each RT-qPCR assay was performed with three biological replicates. All primers are listed in Supplementary Table S7.

### 4.5. Data Analysis

All analyses were performed in triplicate to ensure reliability, and the resulting data were visualized using GraphPad version 10.1.0 (GraphPad, CA, USA), focusing on the mean values for each index. Chord diagram and assessment of correlations between transcription factors and DEGs were generated using Metware Cloud (https://cloud.metware.cn).

## 5. Conclusions

In this study, we observed that the fruiting set rate for self-pollinated ‘Tieton’ (2%) was significantly lower than that for cross-pollinated specimens (56%), thereby confirming that ‘Tieton’ is a self-incompatible variety of sweet cherry. Our transcriptome analysis indicated that the plant-pathogen interaction, plant hormone signal transduction, and plant MAPK signaling pathways were primarily involved in the self-incompatibility response of sweet cherries. Additionally, we identified 13 transcription factors and 132 differentially expressed genes that comprise the core network for self-incompatibility in sweet cherry. In conclusion, these findings provide a theoretical basis for understanding the molecular mechanisms underlying self-incompatibility in sweet cherries and for advancing the breeding of new self-compatible varieties.

## Figures and Tables

**Table 1:** Fruit set rate of self-pollination and cross-pollination in ‘Tieton’.

Pollination Combination	No. of Flowers	No. of Fruits	Fruit Set Rate
‘Tieton’ × ‘Tieton’	50	1	2%
‘Tieton’ × ‘Rainier’	50	28	56%

## Data Availability

The original contributions presented in this study are included in the article/Supplementary Material. Further inquiries can be directed to the corresponding author.

## Supplementary Materials

The following supporting information can be downloaded at: https://www.mdpi.com/article/10.3390/biology14091125/s1, Figure S1: FPKM boxplot for each sample; Figure S2: GO enrichment analysis. (A) CC-12 vs. SI-12; (B) CC-24 vs. SI-24; (C) CC-48 vs. SI-48; Figure S3: KEGG pathway category analysis. (A) CC-12 vs. SI-12; (B) CC-24 vs. SI-24; (C) CC-48 vs. SI-48; Table S1: Summary of the sequencing data quality; Table S2: DEGs identified in different style samples; Table S3: DEGs in plant-pathogen interaction pathway; Table S4: DEGs in plant hormone signal transduction pathway; Table S5: DEGs in plant MAPK signaling pathway; Table S6: Correlation analysis; Table S7. Primers.
