# Supplementary figures and images for: Identification of Self-Incompatibility Related Genes in Sweet Cherry Based on Transcriptomic Analysis
Source: Biology (Basel). 2025 Aug 25;14(9):1125. doi: 10.3390/biology14091125 (PMC12467353; doi:10.3390/biology14091125)

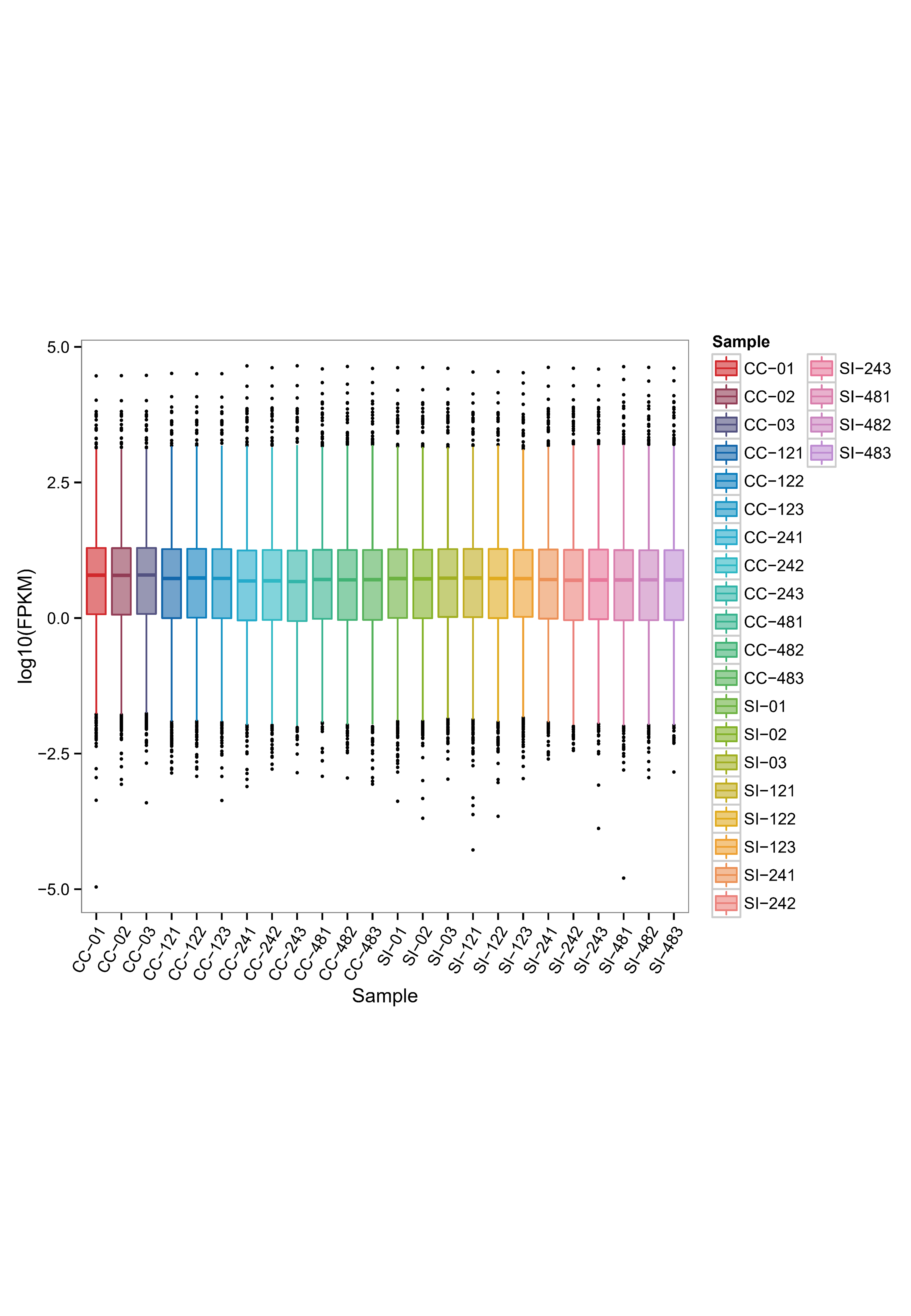

Supplement: Supplementary file 1 [file biology-14-01125-s001.zip › Supplementary Figure S1.TIF]

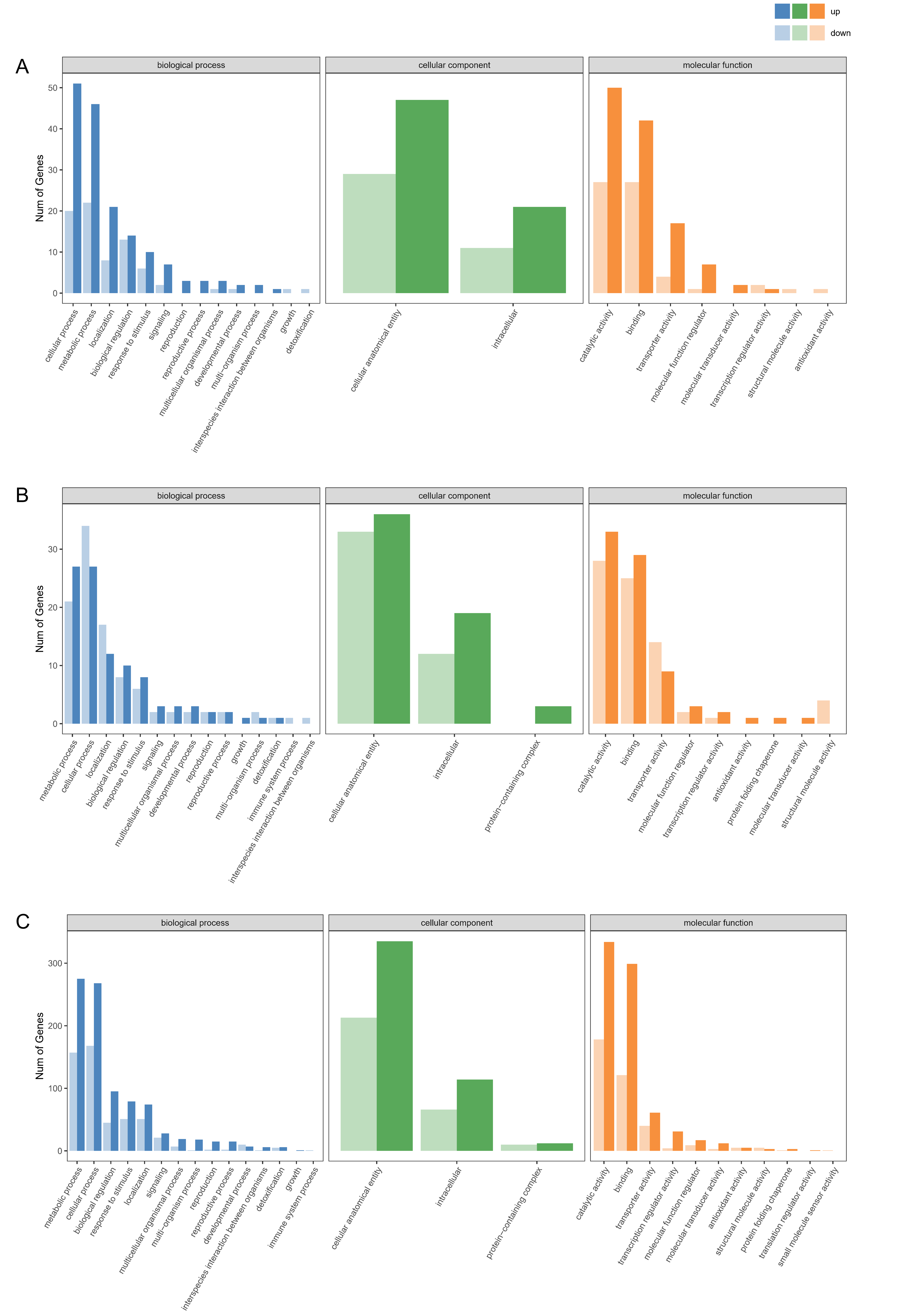

Supplement: Supplementary file 1 [file biology-14-01125-s001.zip › Supplementary Figure S2.TIF]

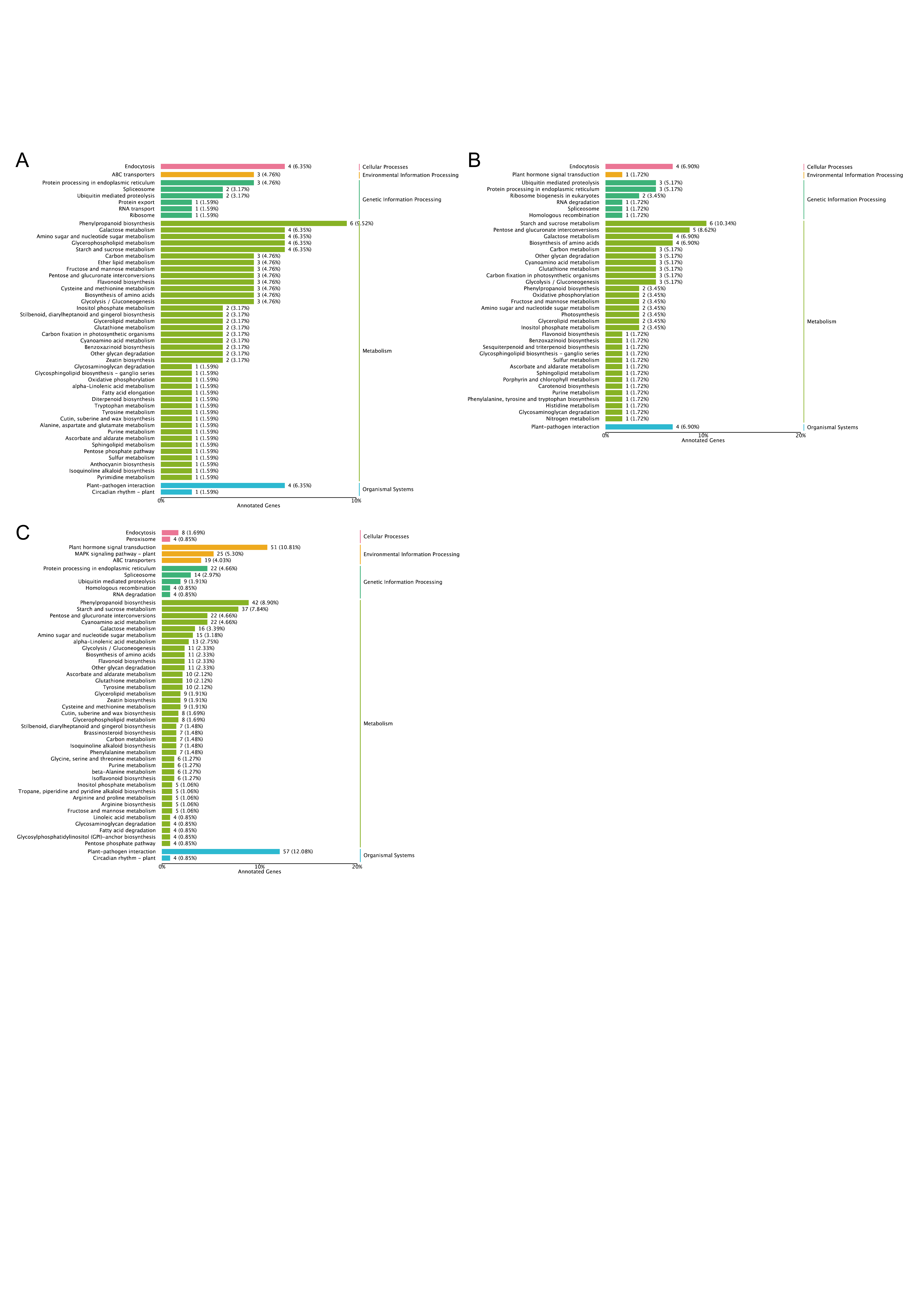

Supplement: Supplementary file 1 [file biology-14-01125-s001.zip › Supplementary Figure S3.TIF]
